# Alcohol Intake Increases in Adolescent C57BL/6J Mice during Intermittent Cycles of Phase-Delayed, Long-Light Conditions

**DOI:** 10.3389/fnbeh.2017.00152

**Published:** 2017-08-21

**Authors:** Joshua J. Gamsby, Abby M. Pribish, Korey D. Stevanovic, Amara Yunus, Danielle Gulick

**Affiliations:** ^1^Byrd Alzheimer’s Institute, University of South Florida Health Tampa, FL, United States; ^2^Department of Molecular Medicine, Morsani College of Medicine, University of South Florida Tampa, FL, United States

**Keywords:** alcoholism, addiction, circadian desynchrony, adolescence, jet lag, phase shift

## Abstract

Adolescents naturally go to bed and awaken late, but are forced to awaken early for school and work. This leads to “social jetlag”, a state of circadian desynchrony (CD), in which internal biological rhythms are out of sync with behavioral rhythms. CD is associated with increased alcohol intake in adults, but has been less well-studied in adolescents. The goal of this study was to model adolescent alcohol intake during similar CD conditions in male C57BL/6J mice. Free access alcohol intake, water intake and wheel-running activity were measured during a normal 12HR photoperiod or during alternating photoperiod (Experiment 1: 12 h light for 4 days followed by 18 h light for 3 days, with dark (activity onset) delayed 9 h during the 18HR photoperiod; Experiment 2: 12 h light for 4 days followed by 6 h light for 3 days, with dark onset delayed 3 h during the 6HR photoperiod). In Experiment 1, CD produced a small but significant increase in the total alcohol intake per day as well as in intake in bouts, with the greatest increase over controls in the hours following the 6HR dark period. Additionally, the pattern of alcohol intake in bouts shifted to increase alcohol intake during the shorter dark period. In Experiment 2, the opposite effect occurred—the longer dark cycle led to lower alcohol drinking in the second half of the dark period. However, in Experiment 2, CD produced no significant changes in either total alcohol intake or alcohol intake in bouts. Conclusion: shifts in the light cycle that disrupt the regular pattern of day and night, and increase the length of the night phase, are sufficient to increase both drinking in bouts and restricted drinking in adolescent mice, modeling increased alcohol intake in adolescents during CD.

## Introduction

Despite the laws, campaigns and preventive efforts of parents and physicians aimed at reducing underage drinking, alcohol abuse remains a major problem among adolescents in the United States. Although consuming alcohol under the age of 21 is illegal in the United States, the CDC reports that adolescents between ages 12 and 20 drink 11% of all alcohol consumed in the country, and over 90% of this is in the form of binge drinking (UDET, 2005). The National Epidemiological Survey on Alcohol and Related Conditions, the broadest of its kind, reported that more than 10% of 11- to 17-year-olds engage in binge drinking (National Institute on Alcohol Abuse and Alcoholism, 2010).

Adolescent alcohol exposure causes significant immediate and long-term consequences to both adolescents and their communities. It correlates with higher rates of academic problems, social problems and reckless behaviors including driving while intoxicated and drug use. For example, a recent national survey reports that underage drinkers were more likely than persons aged 21 or older to report having used illicit drugs within 2 h of alcohol use on their last drinking occasion (19.0 vs. 5.1 percent, respectively; Substance Abuse and Mental Health Services Administration, 2012). Furthermore, adolescent alcohol consumption correlates with alcohol use disorder (AUD) later in life; 45% of adults who began drinking in adolescence meet the criteria for an AUD later in life (Hasin et al., 2007).

The complex etiology of AUD, with multiple susceptibility factors that include a variety of genes and environmental contributions, complicates its diagnosis and treatment. In the adolescent population, the success rate of addiction treatment is particularly low. Treatments for teens are largely ineffective, as behavioral interventions have dropout rates as high as 50% and rates of relapse remain well over 50% (Chung et al., 2004, 2005). Most pharmacotherapy consists of drugs that treat adult alcoholism, and the efficacy and adverse effects of these drugs have not been adequately addressed in adolescents (Liddle and Rowe, 2006; Galanter et al., 2007).

Considering that the large majority of adolescents are naturally night owls but are forced to awaken early for school and work, it is not surprising that a disruption of circadian rhythms is common at this stage of development. Wittmann et al. (2006) have coined the term “social jetlag” for the circadian desynchrony (CD) resulting from social, academic and work schedules, and have demonstrated that this jetlag is associated with increased alcohol and nicotine intake in adolescents (up to age 25). Disruptions in circadian rhythms, through both genetic and environmental mechanisms are more broadly associated with a wide variety of physical, mental and emotional disorders, including substance abuse and dependence (Falcón and McClung, 2009) and have been implicated in the etiology of AUD (Sarkar, 2012). Sleep disruption and changes in circadian gene expression are associated with not only increases in alcohol drinking behaviors, but also increased sensitivity to alcohol (Benca et al., 1992; Wirz-Justice et al., 2001; Perreau-Lenz et al., 2009; Agapito et al., 2010; Comasco et al., 2010; Kovanen et al., 2010). Of special importance to adolescent drinking, Blomeyer et al. (2013) demonstrated that single nucleotide polymorphisms in a key circadian gene, *Period2*, are only associated with high alcohol intake during periods of high environmental stress in 19-year-old men and women, and such stress is common throughout adolescence (Larson and Asmussen, 1991).

Age is a major factor that influences responses to both stress and CD. CD has been shown to impair prefrontal cortical (PFC) function (Karatsoreos et al., 2011). Because the PFC is the center for inhibitory control and is still developing in adolescence, CD may exacerbate susceptibility to alcohol addiction in adolescents. Additionally, CD alters dopamine signaling in the reward circuit (Salgado-Delgado et al., 2011), and CD may impact the already-high sensitivity to alcohol reward in adolescents (Ernst and Fudge, 2009). The fact that the adolescent brain responds differently to substance use likely contributes to the higher rate of AUD in adults who began drinking during adolescence, as early substance use might impair brain development and predispose individuals toward long-term substance dependence.

Although the clinical correlation between CD and alcohol abuse is clear, little work has been done to explore this correlation as it relates to the adolescent brain. In our previous work (Gamsby and Gulick, 2015), a twice-weekly pattern of phase and light cycle shifts increased free access alcohol intake in adult C57BL/6J mice. While this model is applicable to shift workers and frequent travelers, the next step to determine whether we can use the same CD model to examine for the CD experienced by adolescents and young adults, as a first step towards studying how circadian disruption impacts the adolescent brain to increase susceptibility to addiction. The current study assesses changes in alcohol drinking in the adolescent male alcohol-preferring C57BL/6J mouse during a period of repeated phase delays with concurrent changes in the photoperiod.

## Materials and Methods

### Subjects

We used two separate cohorts of mice (*N* = 12 for each experiment) for Experiment 1 and Experiment 2. Male C57BL/6J mice (Jackson Laboratory, Bar Harbor, ME, USA) arrived in the facility and were placed in chambers starting at post-natal day 21. Mice were housed individually in dual lickometer phenotyping chambers (Lafayette Instruments, Lafayette, IN, USA) that measured wheel running, alcohol intake and water intake with* ad libitum* access to food and water. Following the same protocol published previously (Gamsby and Gulick, 2015), a 12-h light:dark cycle (lights on at 7:00) was maintained from this initial habituation through the baseline measurement period. After 4 days of habituation, a second bottle containing 2% alcohol (v/v) was added to each cage. The alcohol concentration was doubled to 4% after 2 days and then doubled again to 8% after 2 more days. Baseline data for analysis began at 33 days old (day 12), 4 days into access to 8% alcohol. Although visible in the longitudinal figures, data from the alcohol escalation period was not examined for this study. The cages were housed in sound-attenuating plant growth incubators (Thermo Scientific, Waltham, MA, USA) that allowed precise control of the luminance and temperature on an hourly and daily basis. All experiments were carried out in accordance with the National Institutes of Health guide for the care and use of Laboratory animals (NIH Publications No. 8023, revised 2011) and were approved by the University of South Florida Institutional Animal Care and Use Committee.

### Experimental Design

#### Experiment 1

In Experiment 1, mice were placed on a 6:00–18:00 photoperiod (18:00–6:00 dark) for baseline. After 7 days of baseline, the dark phase was shortened and delayed by 9 h (3:00–9:00; 18HR light) for half of the mice (*n* = 6; CD). The other half of the mice (*n* = 6; CT) remained on 12:12 LD. After 3 days, the original light phase (6:00–18:00; 12HR light) was restored for 4 days. This was repeated for 21 days in total. The other half of the mice (*n* = 6; CT) remained on a normal 12:12 light cycle. The rationale for the experimental manipulation was to mimic the changes in circadian rhythmicity experienced by adolescents, who frequently go to bed later and sleep longer on the weekends than during the school week.

#### Experiment 2

Experiment 2 followed the same protocol as Experiment 1 except that, following baseline, mice in the experimental condition were exposed to 6 h of light for 3 days followed by 12 h of light for 4 days. After the baseline period, the dark phase was extended and delayed by 3 h (21:00–15:00; 6HR light). After 3 days, the original light phase (6:00–18:00; 12HR light) was restored for 4 days.

### Data Acquisition and Analysis

Data from the lickometers (1 count = 1 lick, or one contact between the mouse’ tongue and the metal tube) and the running wheel (1 count = 1 rotation) were recorded in 5 min bins by a computer running Scurry Activity Monitor Software (Lafayette Instruments, Lafayette, IN, USA). Health checks occurred with the use of infrared goggles during the dark phase of each cycle. At the same time as the health check, the data was downloaded from the computer for later analysis and the alcohol bottles were weighed to measure the volume of alcohol intake over the preceding 24 h. At the end of the experiment, all data were compiled and analyzed using the MATLAB (MathWorks, Natick, MA, USA) software extension CLOCKLAB (Coulbourn Instruments, Whitehall, PA, USA). CLOCKLAB actograms and light/temperature data from HOBO Pendant Temperature/Light Data Loggers (Onset, Cape Cod, MA, USA) were examined to ensure no unexpected spikes in light, temperature, or behavior occurred during the study.

Total alcohol intake (g/kg/day) was calculated using the volume consumed over 24 h (calculated by recording the volume in each bottle at the time of the health check) divided by the body weight at the start of the experimental period. Alcohol intake in bouts (g/kg/min in bouts) was calculated by dividing the volume consumed per day by the number of counts over the prior 24 h to find the volume per count (approximately 1 μL per count), and then multiplying the volume per count by the number of counts in each bout. For bout analysis, we also calculated the peak rate of counts per minute, bout lengths, counts per bout, and bouts per day. Onsets of activity, in which onset was preceded by at least 6 h of inactivity and followed by at least 2 h of activity, alpha, the total activity during the circadian cycle, and the amplitude of the waveform, the difference between the apex and nadir of each activity period, were calculated using the CLOCKLAB software predictive onset analysis, batch analysis and activity profiling, and compared by 2-way repeated measures analysis of variance (RMANOVA). Period was calculated using the CLOCKLAB software, based on the daily activity onsets for each phase of the experiment, excluding the first day of the experimental periods and analyzed by 2-way RMANOVA. All data points were binned by hour or by bout and the following variables were analyzed by RMANOVA or two-way ANOVA: counts per hour, bout length, counts per bout, bouts per day, count rate in bouts, alcohol g/kg intake in bouts and total counts per day. In order to delineate the bouts, they were defined as the period during which there were a minimum 5 counts/min and a maximum gap of 5 min between behaviors. Tukey’s *post hoc* analysis was used to detect significant differences at *p* < 0.05 (SPSS version 23; Chicago, IL, USA).

## Results

### Overall Activity

**Figure 1:**
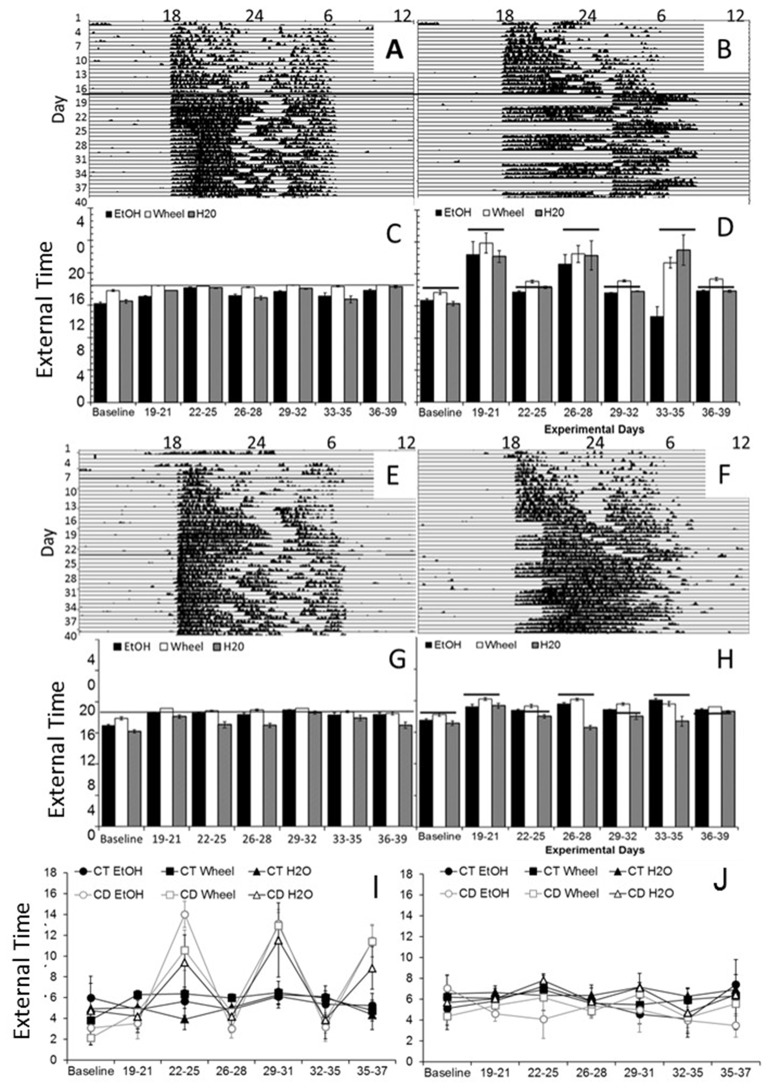
Circadian patterns and period onsets in adolescent C57BL/6J mice during baseline alcohol access and environmental circadian desynchrony (CD). Representative actogram of wheel-running data from two mice in Experiment 1, one in the control condition **(A)**, and one in the CD condition **(B)**. Throughout the study, control mice showed normal circadian rhythms in all three measures **(C)**, with activity onsets at approximately the same time that lights were turned off (18:00, external time). During the CD photoperiods, mice were constantly shifting their behavior onsets to adjust to the phase advances and delays between the 12HR and 18HR photoperiods, respectively **(D)**. Representative actogram of wheel-running data from two mice in Experiment 2, one in the control condition **(E)**, and one in the CD condition **(F)**. Throughout the study, control mice showed normal circadian rhythms in all three measures **(G)**, with activity onsets at approximately the same time that lights were turned off (18:00). During the CD photoperiods, mice were constantly shifting their behavior onsets to adjust to the phase advances and delays between the 12HR and 6HR photoperiods **(H)**. Total activity (alpha) was higher in the mice in the 12HR photoperiod during CD across all measures in Experiment 1 **(I)** but there were no significant effects on alpha in Experiment 2 **(J)** (Data in **C,D** and **G,H** indicate the mean ± SEM for *n* = 6).

In examining overall patterns of activity across the length of the experiment, there were no significant differences in the period length or the time of activity onset between wheel-running, alcohol intake or water intake. During the baseline photoperiod, mice showed normal circadian rhythms in all three measures (mean period: Experiment 1: 24.03, SEM: 0.107; Experiment 2: 23.99, SEM: 0.203). During the CD photoperiods, there were no differences in the length of the period between conditions (mean periods: 23.97–24.05, SEMs: 0.034–0.086). As expected, mice showed no significant daily variations in the time of activity onset during baseline (see Figures 1A–H for representative wheel-running actograms and average onsets), but the phase of onsets oscillated during the CD photoperiods. In Experiment 1, we found a significant difference in onsets between control and CD groups, dependent on the experimental days (*F*_(20,600)_ = 12.49, *p* < 0.001; Figures 1C,D). Unlike in our previous study in adult mice, we found that the mice in the experimental CD condition adapted rapidly to the changing light cycles, and their activity onsets only differed from controls on days 19–21, days 26–28, and days 33–35 (*p* < 0.05 between conditions), suggesting that adolescent mice phase-shifted within approximately one 24-h cycle of the light change. We saw a similar pattern in Experiment 2, with a much smaller effect (*F*_(20,600)_ = 3.17, *p* < 0.01; Figures 1G,H) but significant differences between controls and experimental mice were found on days 19–21, 26–28 and 33–35 (*p* < 0.05). In addition, for all mice, alcohol intake began on average 1.5 h before wheel running (Figures 1C,D,G,H) indicating that the motivation for alcohol may contribute to the onset of activity. CD also caused divergence from predicted activity onsets—control mice initiated wheel-running within 20 min of lights-off throughout the experiment (Figures 1C,G), whereas mice in CD in both experiments became active significantly later in 12:12LD, and significantly earlier in the altered photoperiods (*p* < 0.05, Figures 1D,H), demonstrating that the circadian manipulations were sufficient to shift behavioral rhythms. It is important to note that there are no non-alcohol drinking controls in this study, so we are currently examining whether the rapid adjustment to the phase shifts is affected by chronic exposure to alcohol, an age-dependent effect, or both.

### Activity Patterns

We first examined overall active time (alpha) in each experiment. To do so, we binned data for the last 4 days of baseline, and for each CD period, and then compared the alpha across times and measures (alcohol, wheel and water). For Experiment 1, we found no main or interactive effect of measure, but a significant effect of time-point, *F*_(6,180)_ = 24.49, *p* < 0.001, and of group, *F*_(1,30)_ = 16.14, *p* < 0.001, and an interaction of time-point by group, *F*_(20,42)_ = 8.41, *P* < 0.001. Specifically, independent samples *t-tests* demonstrated that mice in CD had significantly greater active periods during all three 12HR CD photoperiods (*p* < 0.05), but no difference during the 18HR photoperiod—which is striking because they were equally active despite having only half the amount of time in darkness (Figure 1I). For Experiment 2, we found no main or interactive effect of any measure, suggesting that the smaller shift in the photoperiod had no significant effect on activity (Figure 1J).

Next, we examined the waveforms of activity for each measure. Unlike period length and activity onset, which were nearly identical across measures, we observed contrasting patterns of wheel-running, alcohol intake and water intake behaviors over the circadian cycle. In order to compare waveforms more precisely in the 18HR CD condition, we plotted the data to match lights-off (ZT12) across both conditions.

#### Experiment 1

**Figure 2:**
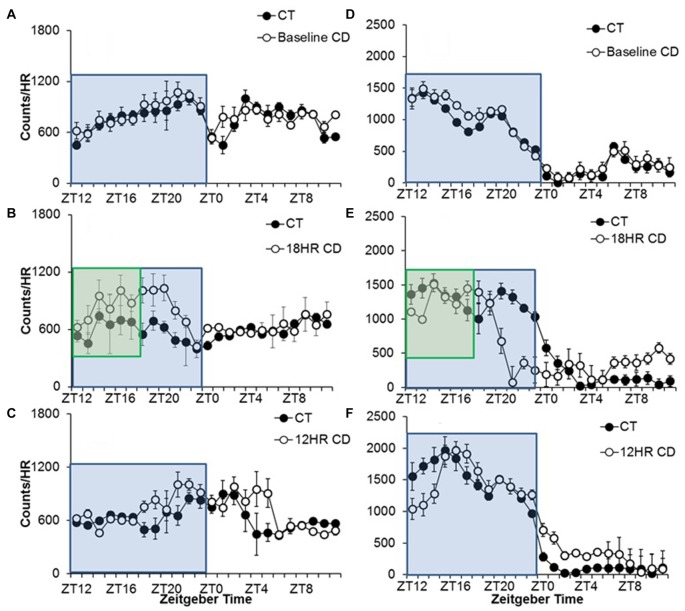
Overall activity counts by hour in adolescent C57BL/6J mice during baseline alcohol access and 18HR environmental CD. There were no differences in patterns of baseline alcohol intake **(A)**, but mice in CD drank significantly more during the 6 h after lights-on in the 18HR photoperiod **(B)** and extended their alcohol intake further into the light cycle in the 12HR photoperiod **(C)**. There were no differences in baseline wheel-running **(D)**, and mice in CD only showed significant activity in the dark (active phase; **E,F**; blue boxes indicate normal 12HR dark; green boxes indicate 6HR dark phase for mice in CD; data indicate the mean ± SEM for *n* = 6).

**Figure 3:**
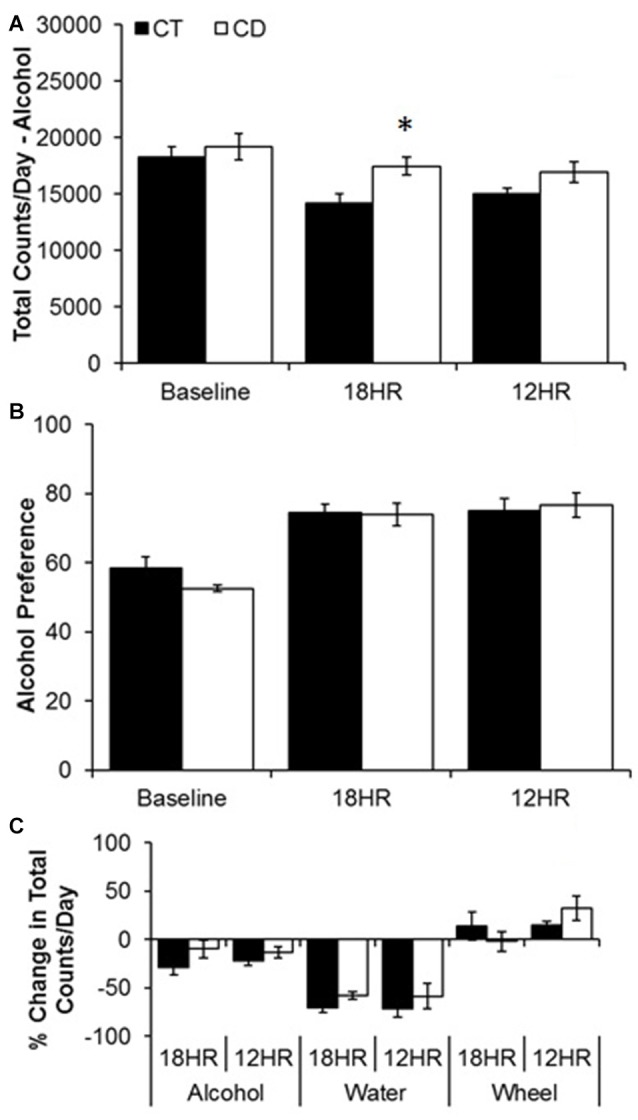
Analysis of overall changes in alcohol and water intake during baseline alcohol access and 18HR environmental CD. Mice in CD drank significantly more alcohol than controls in the total 24-h cycle of 18HR photoperiods (ignoring light and dark; **A**). Although alcohol preference increased over time **(B)** there was no effect of CD and the increase was due to a significant decrease in water intake in both groups **(C)** (data indicate the mean ± SEM for *n* = 6; asterisks indicate significant differences between control and CD groups).

Alcohol drinking varied by hour, *F*_(23,690)_ = 3.47, *p* < 0.001, and the activity by hour was influenced by both photoperiod, *F*_(46,437)_ = 2.79, *p* < 0.01, and condition, *F*_(23,460)_ = 2.23, *p* < 0.05. We also found overall effects of both photoperiod, *F*_(2,32)_ = 6.66, *p* < 0.01, and condition, *F*_(1,33)_ = 7.60, *p* < 0.001 on 24-h alcohol intake. There were no differences in baseline drinking patterns (Figure 2A). Mice in CD drank more total alcohol in the 18HR photoperiod than control mice (Figure 3A), with higher intake from ZT18 to ZT21 (*p* < 0.001; Figure 2B). There was no difference in total intake in the 12HR photoperiod, but mice in CD did drink more alcohol from ZT18 to ZT19, in ZT21, and from ZT4 to ZT5 (*p* < 0.001; Figure 2C). Overall, mice showed an average 5.2% greater amplitude in the waveform of alcohol drinking activity during the 18HR CD periods, with greater alcohol intake during the light phases of CD and with an increase in amplitude in every ensuing 18HR photoperiod (range: 4.2%–7.5%).

Water drinking also varied by hour, *F*_(23,690)_ = 3.09, *p* < 0.001, but there was no effect of photoperiod or condition. The only significant trends were peaks in water intake from ZT4 to ZT7 and at ZT11 and a trough from ZT23 to ZT2 (*p* < 0.05; data not shown). Comparing alcohol and water intake, there was a significant increase in alcohol preference over time, but no differences between groups either across 24 h (Baseline average: 55.5 SEM 5.6; 12HR average: 75.9 SEM 7.4; 18HR average: 74.2 SEM 6.2; Figure 3B) or by hour, and the increase in alcohol preference was due to a ~50% decrease in water intake over time (Figure 3C). This suggests that the mice in CD increased their water intake at the same time that they increased their alcohol intake. While increased water intake would not counter the behavioral effects of alcohol, this finding does raise the possibility that the increased alcohol intake in the CD condition resulted from a generalized increased in fluid consumption.

Wheel-running activity varied by hour, *F*_(23,690)_ = 116.37, *p* < 0.001, and the activity by hour was influenced by both photoperiod, *F*_(46,437)_ = 9.71, *p* < 0.001, and condition, *F*_(23,460)_ = 9.73, *p* < 0.001, although there was no overall difference in total time spent wheel-running between groups in any of the photoperiods. There were no differences during baseline (Figure 2D). Broadly, mice in CD showed lower activity in the 18HR photoperiod in ZT13 and from ZT20 to ZT1, as light suppressed their activity, and a small increase in activity from ZT6-11, in anticipation of the shorter dark cycle (*p* < 0.001; Figure 2E). Mice in CD also showed lower activity in the 12HR photoperiod from ZT12 to ZT14, and greater activity from ZT0 to ZT5 (*p* < 0.001; Figure 2F). The contrast of wheel-running activity only leading up to and in the dark with the increased alcohol intake in the early light period further highlights the fact that the increased alcohol intake in CD is not a general response to CD, but a specific drive to consume alcohol.

#### Experiment 2

**Figure 4:**
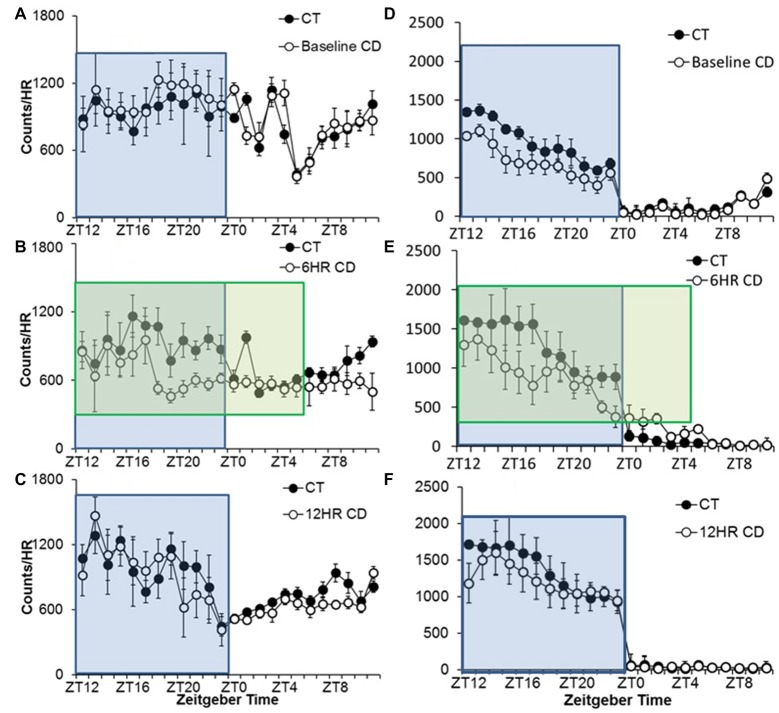
Overall activity counts by hour in adolescent C57BL/6J mice during baseline alcohol access and 6HR environmental CD. There were no differences in patterns of baseline alcohol intake **(A)**, but mice in CD drank significantly less during the second half of the lights-off period in the 6HR photoperiod **(B)** with no changes in the 12HR photoperiod **(C)**. There were no significant differences in baseline wheel-running **(D)**, and mice in CD showed decreased activity in the dark, especially in the 6HR photoperiod (active phase; **E,F**; blue boxes indicate normal 12HR dark; green boxes indicate 18HR dark phase for mice in CD; data indicate the mean ± SEM for *n* = 6).

Alcohol drinking varied by hour, *F*_(23,690)_ = 7.25, *p* < 0.001, and the activity by hour was influenced by both photoperiod, *F*_(46,437)_ = 1.72, *p* < 0.01, and condition, *F*_(23,460)_ = 1.64, *p* < 0.01, but there were no significant differences in 24-h alcohol intake (Table 1). There were no differences in baseline or in the 12HR photoperiod (Figures 4A,C). Mice in CD drank less alcohol from ZT18 to ZT23, in ZT1, and from ZT10 to ZT11 in the 6HR photoperiod (*p* < 0.001; Figure 4B). Overall, the shorter photoperiod produced only a small decrease in alcohol intake in the 6HR photoperiod.

**Table 1:** Analysis of overall alcohol lick counts and bottle preference during baseline alcohol access and 6 h (6HR) environmental CD.

Alcohol	Counts/Day		Preference	
	CT	CD	CT	CD
Baseline	21896.6 (1971)	22428.0 (2983)	55.2 (6.3)	55.0 (3.8)
6HR	20277.1 (1324)	18591.1 (2066)	79.0 (5.0)	77.6 (4.8)
12HR	21155.8 (2157)	20481.1 (2226)	85.9 (3.5)	81.2 (6.6)
			% **CHANGE FROM BASELINE—COUNTS/DAY**	
			**CT**	**CD**
Alcohol	6HR		−8 (4.9)	−20.6 (9.5)
	12HR		−3.5 (4.9)	−9.5 (13.7)
Water	6HR		−70.4 (7.0)	−62.4 (13.8)
	12HR		−80.4 (8.2)	−67.5 (9.5)
Wheel	6HR		29.5 (23.0)	28.8 (4.7)
	12HR		30.6 (1.9)	30.4 (4.5)

*There were no differences in total alcohol intake in any photoperiod (ignoring light and dark; Counts/Day). Although alcohol preference increased over time, there was no effect of CD (Preference) and the increase was due to a significant decrease in water intake in both groups (% Change from Baseline – Water; data indicate the mean (SEM) for *n* = 6)*.

Water drinking also varied by hour, *F*_(23,690)_ = 2.29, *p* < 0.01, with a significant influence of photoperiod, *F*_(46,437)_ = 1.47, *p* < 0.05. However, the only significant finding was a peak in water intake from ZT22 to ZT0 in all photoperiods. The amplitude of the water intake rhythm was reduced by ~50% in the experimental period (*p* < 0.05; data not shown). Comparing alcohol and water intake, there was a significant increase in alcohol preference over time, but no differences between groups, and the increase in alcohol intake was again due to a ~70% decrease in water intake over time (Table 1).

Wheel-running activity varied by hour, *F*_(23,690)_ = 96.08, *p* < 0.001, and the activity by hour was influenced by both photoperiod, *F*_(46,437)_ = 3.69, *p* < 0.001, and condition, *F*_(23,460)_ = 3.80, *p* < 0.001, although there was no overall difference in time spent wheel-running between groups in any of the photoperiods. There were no differences during baseline, despite a trend towards lower activity in the mice that would be exposed to CD (Figure 4D). Broadly, mice in CD showed lower activity in the 6HR photoperiod from ZT16 to ZT17 and ZT22 to ZT23, and greater activity at ZT2 (*p* < 0.001; Figure 4E). Mice in CD also showed lower activity in the 12HR photoperiod in ZT12, but no other differences (*p* < 0.001; Figure 4F).

### Bout Analysis

While overall activity patterns (measured as the average wheel counts/rotations or bottle counts/licks per hour) can help us assess the global effects of CD on behavior, one key aspect of modeling alcohol abuse is the ability to demonstrate changes in the specific pattern of alcohol intake. Thus, we next performed a bout analysis to determine whether behaviors (wheel-running activity, alcohol drinking, and water drinking) were occurring in short, intense periods (bouts) or in longer, more conservative periods. For all analyses, control mice were analyzed across the same time periods as the mice in CD for the sake of comparison, although they remained on the 12:12 LD cycle, and bout data was also analyzed per hour, to control for differences in the lengths of the light cycles.

#### Experiment 1

**Figure 5:**
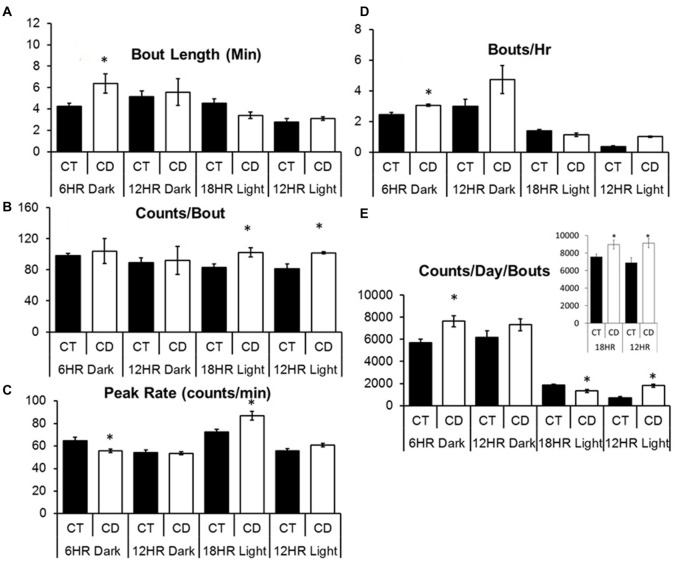
Bout analysis of alcohol drinking by photoperiod in the 18HR environmental CD. Mice in CD had significantly longer bout lengths in the dark in the 18HR photoperiod **(A)**, more counts per bout in the light phase of both 12HR and 18HR photoperiods **(B)**, a lower peak rate in the dark, but higher peak rate in the light phase, of the 18HR photoperiod **(C)**, and more bouts per hour in the dark phase of the 18HR photoperiod **(D)**. In addition, mice in CD had more total counts per day in bouts in both experimental photoperiods as well as in the dark phase of the 18HR photoperiod and in the light phase of the 12HR photoperiod, but fewer counts per day in bouts in the light phase of the 18HR photoperiod **(E)** (data indicate the mean ± SEM for *n* = 6; asterisks indicate significant differences from baseline at *p* < 0.05).

Across conditions, there were significant increases over time in bout length, *F*_(3,32)_ = 3.65, *p* < 0.05, and bouts per day (*F* = 7.11, *p* < 0.001), and decreases in counts per bout (*F* = 3.91, *p* < 0.05), and peak rate (*F* = 11.23, *p* < 0.001), but no overall effect of condition on alcohol drinking. In order to study specific differences during the dark and light periods, we examined dark period and light period bout data separately, and used the CD periods for both control and CD mice in order to make identical comparisons. Mice in CD had significantly longer bout lengths in the 6HR dark period, *t*_(1,10)_ = 2.50, *p* < 0.05; Figure 5A) compared to controls in the same period, more counts per bout in the total 18HR CD period (*T* = 2.74, *p* < 0.05; CT = 87.6 SEM 3.4; CD = 106.5 SEM 5.7), as well as in both the 18HR (*T* = 2.56, *p* < 0.05; Figure 5B) and 12HR (*T* = 2.24, *p* < 0.05) light periods, lower peak rate in the 6HR dark period (*T* = 2.48, *p* < 0.05) but higher peak rate in the 18HR light period (*T* = 2.85, *p* < 0.05; Figure 5C). Mice in CD had a small but significant increase in bouts per hour in the 6HR dark period (*T* = 3.51, *p* < 0.01; Figure 5D) as well as a trend toward more bouts per hour in the 12HR dark period (*p* = 0.058). Finally, mice in CD had more counts per day in bouts in the total 12HR (*T* = 2.45, *P* < 0.05; Figure 5E, inset) and 18HR CD periods (*T* = 2.27, *P* < 0.05), as well as in the 6HR dark period (*T* = 3.03, *p* < 0.01; Figure 5E) and 12HR light period (*T* = 2.88, *p* < 0.05), but less in the 18HR light period (*T* = 2.06, *P* < 0.05). Although there was no consistent change across the different light and dark periods, mice in CD did change their behavioral patterns to increase total drinking in bouts, as demonstrated by the increased counts per day in bouts, and especially by the fact that there was an increase in total counts per day in bouts in the 18HR light phase of CD, when the mice should have been sleeping significantly more. It is also important to note that the drinking in bouts only accounted for approximately 1/2 of total alcohol intake, based on the number of counts per day in bouts vs. total counts per day. This suggests that a significant amount of alcohol drinking occurred in restricted (outside of bouts) alcohol intake in all of the mice.

For total alcohol intake (g/kg), there was a significant effect of time (*F*_(2,18)_ = 8.76, *p* < 0.05), and a significant effect of condition (*F*_(1,9)_ = 5.1, *p* < 0.05), but no interaction. Total alcohol intake decreased over time (*p* < 0.05; Table 2). Individual *t*-tests demonstrated that the mice in CD were only significantly higher than controls in the 12HR photoperiod. For alcohol intake per minute in bouts, there was a significant effect of time (*F*_(2,18)_ = 13.65, *p* < 0.001), and a significant interaction of time and condition (*F*_(2,18)_ = 4.16, *p* < 0.05) with intake per bout decreasing over time, but the only significant difference between groups was at baseline. For alcohol intake per day in bouts, there was a significant effect of time (*F*_(2,18)_ = 7.14, *p* < 0.01), and a significant interaction of time and condition (*F*_(2,18)_ = 3.16, *p* < 0.05) with alcohol intake in bouts increasing over time and significantly higher intake in the mice in the 12HR CD photoperiod. While we were unable to assess blood alcohol levels without disturbing the circadian rhythms of the mice, the volume of alcohol consumed in the bouts is comparable to the volumes consumed in other studies of alcohol intake in mice, as discussed below.

**Table 2:** Analysis of alcohol intake in bouts (g/kg/min and total g/kg) and over 24 h (g/kg), based on photoperiod in Experiment 1.

Alcohol intake (g/kg)	Per bout		Per day in bouts		Total per day	
All data: mean (SEM)	CT	CD	CT	CD	CT	CD
Baseline	0.088 (0.007)	0.11 (0.009)*	4.34 (0.856)	4.93 (0.210)	17.11 (0.860)	17.95 (1.190)
12HR light	0.082 (0.004)	0.100 (0.006)	6.42 (0.730)	8.38 (0.310)*	13.30 (0.752)	16.35 (0.797)*
18HR light	0.083 (0.005)	0.089 (0.001)	7.07 (0.736)	8.53 (0.414)	14.04 (0.463)	15.82 (0.930)

*There was a small but significant difference in alcohol intake per bout during baseline. However, the only differences in alcohol intake in bouts or overall per day was in the 12HR light period of the experimental phase (including both light and dark phases of this period; (data indicate the mean (SEM) for *n* = 6; asterisks indicate significant differences from controls at *p* < 0.05)*.

Across conditions, there were significant increases over time in bout length, *F*_(3,32)_ = 9.86, *p* < 0.001, counts per bout (*F* = 5.80, *p* < 0.01), counts per day (*F* = 3.52, *p* < 0.05), and peak rate (*F* = 7.46, *p* < 0.001), but decreases in bouts per day (*F* = 12.03, *p* < 0.001), but no overall effect of condition on wheel running. In order to study specific differences during the dark and light periods, we examined dark period and light period bout data separately. Mice in CD had significantly shorter bout lengths in the 18HR light period *T*_(1,10)_ = 2.42, *p* < 0.05; Table 3) compared to controls in the same period, and significantly more bouts per hour in the 6HR dark period (*T* = 6.95, *P* < 0.01; Table 3) and 12HR dark period (*T* = 4.98, *p* < 0.05), but no differences in counts (Table 3), bouts per day, or peak rate (Table 3). When contrasted with the alcohol drinking data, this demonstrates that the increased alcohol intake in bouts was not due to a general increase in waking or activity, as the mice in CD only showed increases in bouts per hour in the dark cycle.

**Table 3:** Bout analysis of wheel-running by photoperiod in the 18HR environmental CD.

	6HR dark		12HR dark		18HR light		12HR light	
	CT	CD	CT	CD	CT	CD	CT	CD
Avg bout length	18.2 (3.6)	29.2 (9.3)	31.2 (7.0)	36.1 (8.5)	34.8 (3.9)	24.8 (4.7)*	27.6 (5.7)	9.1 (8.4)
Avg counts/bout	453.8 (79.0)	1079.8 (356.6)	955.4 (340.0)	1440.2 (295.3)	989.5 (174.6)	847.5 (150.0)	705.8 (265.5)	490.7 (307.0)
Avg peak rate	42.4 (4.9)	49.9 (7.8)	46.9 (5.8)	53.0 (8.0)	44.3 (4.6)	53.0 (2.9)	40.6 (5.0)	38.9 (2.7)
Bouts/h	3.4 (0.6)	7.0 (0.4)*	3.9 (0.5)	7.5 (1.5)*	0.2 (0.2)	1.0 (0.1)	0.3 (0.2)	0.1 (0.1)

*There were no differences in the total counts on the wheel between photoperiods, nor in counts per bout or in peak rate, but mice in CD did have shorter bout lengths in the light phase of the 18HR photoperiod and more bouts per hour in the dark phase of both 12HR and 18HR photoperiods (data indicate the mean (SEM) for *n* = 6; asterisks indicate significant differences from controls at *p* < 0.05)*.

In the bout analysis of water intake, there was a change over time in bout length, *F*_(3,32)_ = 9.86, *p* < 0.001 (increases only in experimental weeks 1 and 2), counts per day (*F* = 3.52, *p* < 0.05; increases only in experimental weeks 1 and 2), and an interaction of time and condition on peak rate (*F*_(3,32)_ = 3.57, *p* < 0.05; rate only increased in the mice exposed to CD). In order to study specific differences during the dark and light periods, we examined dark period and light period bout data separately. Mice in CD had significantly more bouts per hour in the 6HR dark period (*T*_(1,10)_ = 6.95, *P* < 0.01; Table 4) and 12HR dark period (*T* = 4.98, *p* < 0.05) but no significant difference in bout length, peak rate, counts per bout (Table 4), or in bouts per day. When contrasted with the alcohol drinking data, although there were increases in bouts per hour in water drinking during the dark periods of CD, the bout lengths, peak rates and counts/bout were much higher in alcohol drinking, indicating the higher preference and activity for alcohol and, again, mice in CD only showed increases in bouts per hour during their dark cycle.

**Table 4:** Bout analysis of water drinking by photoperiod in the 18HR environmental CD.

	6HR dark		12HR dark		18HR light		12HR light	
	CT	CD	CT	CD	CT	CD	CT	CD
Avg bout length	2.4 (0.2)	2.4 (0.5)	2.2 (0.3)	2.5 (0.5)	3.1 (0.3)	3.2 (0.2)	2.1 (0.3)	2.0 (0.3)
Avg counts/bout	39.6 (7.6)	40.4 (8.0)	31.2 (4.0)	40.4 (7.1)	43.9 (3.6)	58.11 (2.4)	30.6 (3.8)	42.0 (4.5)
Avg peak rate	24.9 (3.5)	29.2 (2.7)	22.8 (1.8)	28.7 (0.8)	30.0 (2.0)	42.0 (1.3)	22.8 (1.8)	30.78 (2.3)
Bouts/hr	1.9 (0.4)	4.9 (0.1)*	2.0 (0.2)	4.8 (0.4)*	1.1 (0.1)	0.6 (0.1)	0.1 (0.2)	0.4 (0.1)

*There were no differences in the total counts of water intake between photoperiods, nor in bout lengths, counts per bout or in peak rate, but mice in CD did have more bouts per hour in the dark phase of both 12HR and 18HR photoperiods (data indicate the mean (SEM) for *n* = 6; asterisks indicate significant differences from controls at *p* < 0.05)*.

#### Experiment 2

Across conditions, there were significant increases over time in alcohol drinking counts per bout, *F*_(3,32)_ = 3.56, *p* < 0.05 (only in experimental week 2), and bouts per day (*F* = 7.11, *p* < 0.001), but no change in any other measure and no overall effect of condition on alcohol drinking (data not shown). Further, mice in CD did not differ from control mice in any of the other bout analysis measures that we examined (Table 5).

**Table 5:** Bout analysis of alcohol drinking by photoperiod in the 6HR environmental CD.

	18HR dark		12HR dark		6HR light		12HR light	
	CT	CD	CT	CD	CT	CD	CT	CD
Avg bout length	8.5 (0.4)	8.6 (1.4)	7.0 (0.3)	7.3 (1.4)	8.5 (0.5)	9.1 (1.1)	8.9 (0.4)	9.4 (1.5)
Avg counts/bout	116.0 (5.6)	135.0 (14.0)	120.8 (10.8)	133.4 (17.6)	138.8 (11.4)	132.5 (17.5)	122.9 (6.9)	122.7 (16.2)
Avg peak rate	65.5 (0.8)	68.6 (2.0)	79.6 (1.6)	86.1 (1.7)	79.7 (1.5)	71.2 (2.7)	67.1 (0.8)	63.0 (1.8)
Bouts/h	0.8 (0.03)	0.6 (0.03)	0.7 (0.04)	0.6 (0.1)	2.5 (0.1)	2.9 (0.1)	2.5 (0.1)	2.4 (0.2)

*There were no differences in the total counts on the wheel between photoperiods, nor in bout lengths, counts per bout, peak rate, or in bouts per hour (data indicate the mean (SEM) for *n* = 6)*.

## Discussion

This study demonstrates that CD is sufficient to produce a small but significant increase alcohol intake in bouts by adolescent C576BL/6J mice, as well an overall increase in restricted alcohol drinking. All adolescent mice decreased their total alcohol intake per day over the course of the study, but increased their alcohol intake in bouts, establishing more intense drinking patterns. In response to CD, the adolescent mice drank longer into their inactive (light) period in both the normal (12:12) and altered (18:6) photoperiods, although the effect was more pronounced in the 18HR light period. This change in alcohol drinking occurred independently of changes in wheel-running. One caveat in our results is the concurrent increase in water intake during high alcohol intake, which produced a null effect on alcohol preference. Thus, mice may have consumed more alcohol as a side effect of heightened fluid consumption. Nonetheless, the increase in water intake would not counter the physiological and behavioral effects of alcohol, and the CD mice still achieved greater alcohol intake levels. Ongoing studies are examining how CD affects alcohol conditioned place preference and alcohol sensitivity in order to parse out the factors contributing to heightened alcohol intake in this study.

Because we use a free access model in order to assess 24-h patterns of alcohol intake, our findings demonstrate a self-selected increase in alcohol-seeking behavior during CD. The effect of CD on alcohol intake in this study supports this model for the effect of CD on alcohol and substance abuse in human adolescents. In addition, since human and rodent (PND 21–60) adolescents undergo similar milestones in terms of cortical development and changes in motivation, impulsivity, and affect (Spear, 2000; Crews et al., 2007), we are now using this model to examine the underlying neural changes that lead to enhanced alcohol intake in CD.

The shift toward higher alcohol drinking in adolescent mice in response to CD is similar to the process of self-medication with alcohol that has been documented in adolescents in response to CD (Shibley et al., 2008). This study suggests that changes in circadian cycles in adolescents, whether because of academic, peer, or family pressures, can result in behavioral compensation, including increased substance use. The finding that regular changes in photoperiod correlate with a shift toward drinking in bouts aligns with research in humans showing that a greater imbalance between weekday and weekend sleep schedules is also associated with alcohol intake in adolescents (O’Brien and Mindell, 2005), which strongly implicates CD in adolescent alcohol abuse. Additionally, the photoperiod manipulation in this study (a sequence of phase delays followed by restoration of the original 12HR light phase) mimics the typical weeklong cycle of increased adolescent CD during the workweek with a return to a more natural circadian cycle during the weekend. Surprisingly, the mice in CD were significantly more active in the 12HR photoperiods, suggesting increased activity to compensate for the longer inactive phase of the 18HR photoperiod—yet they were not significantly less active in that 18HR photoperiod. This suggests that our CD protocol produced a maladaptive shift in overall activity, which could contribute to fatigue and stress. The waveform (peak) in alcohol intake also increased during every ensuing 18HR photoperiod, suggesting an escalating maladaptive increase in alcohol intake in these periods. One interesting outcome of this study is that the adolescent mice rapidly reset their circadian behavior to match the new photoperiods, unlike adult mice. Considering that the master circadian clock in the suprachiasmatic nucleus develops between embryonic day 12 and postnatal day 14 (Landgraf et al., 2014), it is unlikely that plasticity in this region underlies the age-dependent differences in our findings. However, a recent study demonstrated that adolescent mice differ from adult mice in their endogenous rhythmicity and in their sensitivity to alcohol effects on entrainment, and we are currently investigating this phenomenon further.

Overall, adolescent mice showed a greater amplitude oscillation in alcohol drinking in the 18HR photoperiod and a broader peak in the 12HR photoperiod compared with both controls and baseline photoperiods, with greater alcohol intake during the light phase. However, we found that adolescent mice increase their drinking in bouts, while decreasing total intake over time, and CD produced only small changes in alcohol intake in bouts in adolescents, but significantly increased total alcohol consumed. In ongoing experiments, we are assessing the differences in how the adolescent brain and adult brain respond to CD and other stressors, with a focus on biomarkers of stress and addiction.

Observing the correlation between CD and alcohol consumption patterns in adolescence is an important first step in understanding the environmental and genetic contributions to early alcohol abuse, and a prerequisite to future efforts to clinically address substance use in this population. Current behavioral interventions and pharmacotherapy for substance abuse have been largely ineffective and inadequately studied in the adolescent population (Chung et al., 2004, 2005; Liddle and Rowe, 2006; Galanter et al., 2007). Ideally, environmental CD could be corrected by changing work or school schedules, but since this is rarely an option, future studies will aim to identify potential targets in these pathways for therapeutics to treat AUD during CD. We are focusing on identifying the mechanisms by which CD causes a shift in alcohol intake in adolescents, as well as examining the role of sex and hormones in these interactions.

In conclusion, this study demonstrates that CD induces a self-selected shift toward alcohol drinking in both bouts and in restricted drinking in adolescent male mice. Further studies will assess the mechanisms by which environmental CD induces changes in alcohol drinking patterns, with an ultimate goal of targeting appropriate behavioral interventions and pharmacotherapy for substance abuse toward adolescents experiencing CD.

## Author Contributions

DG designed all studies, ran all statistics and wrote the final article. AY, KDS and AMP ran the day-to-day experimentation and collected the data and AMP also assisted in drafting the manuscript. JJG provided the circadian set-up for all experiments, edited the manuscript and also consulted on the correct circadian analyses to be completed.

## Conflict of Interest Statement

The authors declare that the research was conducted in the absence of any commercial or financial relationships that could be construed as a potential conflict of interest.
